# Variability of the Prevalence of Depression in Function of Sociodemographic and Environmental Factors: Ecological Model

**DOI:** 10.3389/fpsyg.2018.02182

**Published:** 2018-11-12

**Authors:** José María Llorente, Bárbara Oliván-Blázquez, María Zuñiga-Antón, Bárbara Masluk, Eva Andrés, Javier García-Campayo, Rosa Magallón-Botaya

**Affiliations:** ^1^Health Research Institute of Aragon, Zaragoza, Spain; ^2^Aragones Health Service, Zaragoza, Spain; ^3^Department of Psychology and Sociology, University of Zaragoza, Zaragoza, Spain; ^4^Primary Care Prevention and Health Promotion Network (RedIAPP), Madrid, Spain; ^5^Department of Geography and Territorial Planning, University of Zaragoza, Zaragoza, Spain; ^6^Department of Applied Economics, Autonomous University of Madrid, Madrid, Spain; ^7^Department of Medicine and Psychiatry, University of Zaragoza, Zaragoza, Spain

**Keywords:** depression, variability, prevalence, sociodemographic factors, environmental factors

## Abstract

Major depression etiopathogenesis is related to a wide variety of genetics, demographic and psychosocial factors, as well as to environmental factors. The objective of this study is to analyze sociodemographic and environmental variables that are related to the prevalence of depression through correlation analysis and to develop a regression model that explains the behavior of this disease from an ecological perspective. This is an ecological, retrospective, cross-sectional study. The target population was 1,148,430 individuals over the age of 16 who were registered in Aragon (Spain) during 2010, with electronic medical records in the community’s primary health care centers. The spatial unit was the Basic Health Area (BHA). The dependent variable was the diagnosis of Depression and the ecological independent variables were: Demographic variables (gender and age), population distribution, typology of the entity, population structure by sex and age, by nationality, by education, by work, by salary, by marital status, structure of the household by number of members, and state of the buildings. The results show moderate and positive correlations with higher rates of depression in areas having a higher femininity index, higher population density, areas with a higher unemployment rate and higher average salary. The results of the linear regression show that aging +75 and rural entities act as protective factors for depression, while urban areas and deficient buildings act as risk factors. In conclusion, the ecological methodology may be a useful tool which, together with the statistical epidemiological analysis, can help in the political decision making process.

## Introduction

Depression is a highly prevalent mental disorder in our society, with 10–20% of the population experiencing a depressive disorder during their lives (Üstün et al., 2004; Bromet et al., 2011; Gutiérrez-Fraile et al., 2011). Depression increases the risk for early mortality and has become the second most common cause of disease-induced disability in our developed society (Gabilondo et al., 2010; Stegmann et al., 2010; Ferrari et al., 2013). In addition, it is the most expensive mental disorder in Europe, representing 1% of the total European economy (Sobocki et al., 2006; Mykletun et al., 2009).

Major depression etiopathogenesis is related to a wide variety of genetic (Cervilla et al., 2006, 2007) demographic and psychosocial factors (Patten et al., 2009; Bellón et al., 2011; Hidaka, 2012; Kupfer et al., 2012) as well as to environmental factors (Probst et al., 2006; Annequin et al., 2015; O’Hare et al., 2016). The most frequently studied sociodemographic factors, related to depression are: gender (Weissman and Olfson, 1995; Aragonès Benaiges et al., 2003; Barry et al., 2008); age (Roberts et al., 1997; Weissman and Wickramaratne, 2000; Gum et al., 2009; Kessler et al., 2010; Trainor et al., 2013); ethnic group (Somervell et al., 1989; Kessler, 2003) cultural and educational level (Kempen et al., 1999; Koster et al., 2006; Weich et al., 2007; Bjelland et al., 2008); marital status and relationship (Whisman and Bruce, 1999; Lorant et al., 2007; Plaisier et al., 2008) social relationship and social support (Stansfeld et al., 2003; Leskela et al., 2004; Brugha et al., 2005; Kendler, 2005; Golden et al., 2009; Lino et al., 2013); and socioeconomic level and unemployment (Weich and Lewis, 1998; Lorant et al., 2003, 2007). Other analyzed risk factors are comorbidity with other physical and psychiatric diseases (Sekula et al., 2003; Djernes, 2006), disability (Spijker et al., 2004), experiences of discrimination (Karlsen et al., 2005) and abuse in childhood (Rohde et al., 2008), work demands (Plaisier et al., 2007), stressful life events (Patton et al., 2003; Bos et al., 2007) etc.

In response to these researches, a scarce bibliography exists, that delves into the association between environmental factors and the prevalence and/or etiopathogenesis of depression. The most frequently studied environmental factors have been the influence of the rural or urban residence, the proximity to natural spaces or social interaction, physical environment, climatology, or changes in temperatures. Regarding the rural/urban residence, several studies state that depression rates in rural areas are lower once adjusting for confounding factors (Wang, 2004; Weich et al., 2006). This may be explained by a higher sense of belonging to their community and higher social support (Romans et al., 2011). As for climatology, a relationship has been found between climatic factors such as ambient temperature, light duration and changing and extreme climates, and depression (Jessen et al., 1998; Gaxiola-Robles et al., 2013); other factors such as a favorable environment and the proximity to natural spaces have been related to a better well-being and mood (Regan and Horn, 2005; Ryan et al., 2010; Berman et al., 2012). However, this bibliography does not analyze collective environmental factors and there are few studies that address them from an ecological perspective.

The objective of this study is to analyze sociodemographic and environmental variables that are related to the prevalence of depression through correlation analysis and to develop a regression model that explains the behavior of this disease from an ecological perspective. The hypothesis of this study is that sociodemographic variables are related with the prevalence of depression, with different strength, and direction, as protector or risk factors.

## Materials and Methods

### Design

This is an ecological, retrospective, cross-sectional study.

### Setting and Study Population

The target population was 1,148,430 individuals over the age of 16 who were registered in Aragon (Spain) during 2010, with electronic medical records in the community’s primary health care centers. The autonomous community under study has an area of 47,719 square Km and a population density of 28.20 inhabitants per square kilometer. Its population is over-aged, which is more concentrated in rural areas, with the capitals having a younger population structure. The capital of the community (Zaragoza) contains half of the population and there are only thirteen municipalities that exceed 10,000 inhabitants. Rural nuclei (with less than 2000 inhabitants) represent 86% of the municipalities, where only 16.8% of the population lives.

**FIGURE 1:**
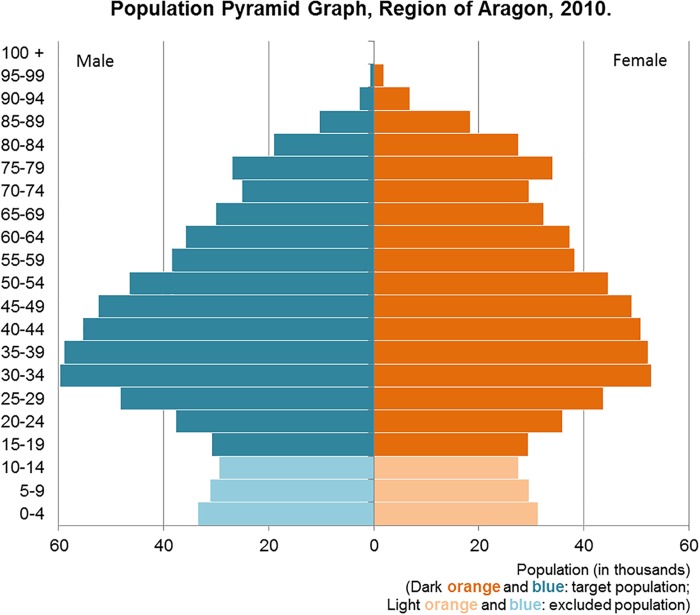
Population Pyramid Graph. Region of Aragon. 2010.

The population pyramid graph related to Aragon, in Figure 1, shows a contracting structure. On one hand, the main groups by age are 30–49 age people (active population), this age group has been increased by the significant arrival of immigrant population since 2000. On the other hand, the importance of the population over 65 is growing significantly since the beginning of the century. This is a common feature in all developed regions and an element to be taken into account in government and health policies. The origin of this situation is double: an important decrease of fertility rates in the region and an increase of Life expectancy at birth, especially in the older cohorts. The pyramid allows to see that Crude birth rates are low, because the first cohorts (0–4 years old) are a low number, and Crude death rates are low, also.

The sample of this study consists of all individuals having open electronic medical records in health centers of the autonomous community of Aragon (Spain), for at least 2 years during the time of entry into the study, including patients with an active diagnosis of depression during the year of the study. Records containing inconsistencies in the database were excluded from the study. These were the cases of patients with diagnosis of death and those revealing less than one doctor’s visits during the year of analysis.

Due to the universal nature of the health system and the absence of other primary health care providers, the data obtained in the study is considered to be representative of practically 100% of the population that met the criteria for inclusion in the study.

### Outcomes and Instruments

The spatial unit in which both the dependent and independent variables were correlated was the Basic Health Area (BHA).

The dependent variable of the study was the diagnosis of Depression, for which the International Classification in Primary Care (ICPC) of the World Organization of Family Doctors (WONCA) was used. This data was collected from electronic medical records of all primary care patients in the community.

The ecological independent variables of the study were related to the prevalence of depression, which have been found in previous studies in the literature consulted. These variables were: Demographic variables (gender and age), population distribution, typology of the entity, population structure by sex and age, by nationality, by education, by work, by salary, by marital status, structure of the household by number of members, and state of the buildings. For each of these variables, the source of information, the scale of work, and the indicators used are indicated below, in Table 1. These variables were collected from the data for the year 2010.

**Table 1:** Variables of the study, indicators, sources, and units of measure.

	Variables	Indicators	Source	Unit of measure
Dependent Variable				
	Prevalence of depression by BHA		Electronic medical records	Percentage
Independent Variables				
Demographic variables	Gender and age		Electronic medical records	Percentage
Population distribution and typology of entity	Population distribution	Total population	SNIS	Total number
		Density of population	SNIS	inhab/km^2^
	Typology of entity	Type of settlement	ASI	Categories
Demographic characteristics	PS by gender and age	Femininity index	SNIS	Percentage
		Dependence index	SNIS	Percentage
		Index of over-aging	SNIS	Percentage
Socioeconomics characteristics	PS by nationality	Rate of foreigners	SNIS	Percentage
	PS by education	Average education degree	ASI	Average number
	PS by work	Unemployment rate	AEI	Percentage
	PS by salary	Average salary	TA	Euros
	PS by marital status	Single population rate	ASI	Percentage
		Separated, divorced, or widowed population rate	ASI	Percentage
Characteristics of the home, housing, and building	Household structure by number of members	Unipersonal household	ASI	Percentage
		Households of 5 or more members	ASI	Percentage
	State of the buildings	Poor or worse state	ASI	Percentage
		Bad or worse state	ASI	Percentage

*BHA, basic health area; PS, population structure; SNIS, spanish national institute of statistics; ASI, aragon statistics institute; AEI, aragon employment institute; TA, tax agency*.

Analyzed demographic variables were gender and age, collected from electronic medical records.

The population distribution variable was measured with the following indicators: (1) total population, measured by total number; and (2) population density, measured by inhabitants per square kilometer^71^. This variable was collected according to the official figures of Population of Spanish Municipalities of the Spanish National Institute of Statistics (SNIS).

The Typology of entity variable, following the standard classification collected by the Aragon Statistics Institute (ASI, 2010), was classified as Rural zone: made up of municipalities of up to 2,000 inhabitants; intermediate zone: made up of municipalities from 2,001 to 10,000 inhabitants; and urban area: made up of municipalities with over 10,000 inhabitants. This variable was collected from the official figures of the Aragon Statistics Institute (ASI, 2010).

The variable of population structure by sex and age was measured based on the following indicators: (1) femininity index that is expressed as the number of women per 100 men, expressed as a percentage; (2) dependence index indicating the number of people under 15 and over 64 years old per 100 people between the ages of 15 and 64. It is expressed in percentage; and (3) index of over-aging (75 and 85), which indicates the number of people aged 75 years old and over; and of 85 years old and over, per 100 people aged 65 years and over (Reques Velasco, 2010). These indicators are expressed in percentages. They were obtained from the Official Population Figures from Spanish Municipalities compiled by the SNIS.

The Structure of the population by nationality variable was measured using the rate of foreigners, defined as the number of foreigners out of the total population of a BHA and expressed as the number of foreigners per 100 individuals (percentage). These data were obtained from the Official Population Figures from Spanish Municipalities compiled by the Spanish National Institute of Statistics (SNIS, 2010).

The Structure of the population by education variable was measured using the average grade of education indicator, which is the sum of class marks of the level of studies of people aged 16 and over divided by the total number of people of those ages residing in the studied territory. The class mark is the half-sum of the extremes of the interval and represents a central value of it. It is expressed as a value between 1 and 4 corresponding to (1) Without studies, (2) First grade, (3) Second grade and (4) Third grade. It represents a mean value. These data were obtained from the Population and Housing Censuses of the ASI.

The Structure of the population by work variable was collected using the unemployment rate as an indicator, which is defined as the percentage of the population in an unemployment situation with respect to the total population between 16 and 65 years of age. It is expressed as a percentage. These data were obtained from the Labor, Wages and Labor Relations Statistics of the Aragon Employment Institute (AEI, 2010).

The Structure of the population by salary variable was recollected using the Average Salary as an indicator, which is considered the average salary of each inhabitant in thousands of euros, understood as the relationship between perceptions, and payments. It is therefore quantified in euros. These data were obtained from the labor market database and pensions in the tax sources (Tax Agency based on the Annual Withholding Tax on Labor Income Tax Return (Tax Agency, 2010).

The Structure of the population by marital status variable, was collected according to the following indicators: (1) Single Population rate, understood as the ratio between the number of people with single marital status and the total number of people over 16 years of age; and (2) the rate of a separated, divorced or widowed population, understood as the ratio between the number of persons with a personal status of separated, divorced, or widowed and the total number of persons over 16 years of age. Both indicators are expressed in percentages. The rate of married population was not considered as being complementary to the analyzed rates and as a protective factor against depression, especially in men (Plaisier et al., 2008). This variable was collected from the Population and Housing Census, of the ASI.

The Household structure by number of members variable was collected using the following indicators: (1) unipersonal households, understood as the relationship between the number of households of a single member and the total number of households; and (2) households of 5 or more members, understood as the ratio between the number of households composed of 5 or more members and the total number of households. Both indicators are expressed in percentages. These data were obtained from the Population and Housing Censuses of the ASI.

The state of the buildings variable was collected using the following indicators: (1) Poor or worse state of the buildings, which is the percentage of buildings that, in the last census, were classified as a decaying, bad, or deficient building in relation to the total of buildings, and (2) Buildings in bad or worse condition, which is the percentage of buildings that were classified as decaying or bad in relation to the total number of buildings in the last census. The data was obtained from the Population and Housing Censuses of the ASI.

Accessibility to equipment and services has not been included as a variable in the analysis, since these data were not available for the entire autonomous community.

### Statistical Analysis

The unit used to correlate and analyze the studied variables is the BHA, as a delimitation of the territorial framework in which the primary care professionals act. The average population of a BHA in Aragon is 10,622.01 inhabitants (SD: 8,762.522).

For the representation of the initial descriptive study in the model, direct standardization is carried out on the cases observed by age and sex. Age and sex are shown as two confounding factors in relation to morbidity, as confirmed by the literature (Kessler, 2003; Urbina Torija et al., 2007). In order to control their effect, specific prevalence rates are calculated by groups of sex and age by BHA with Epidat 3.1^®^.

From an ecological epidemiological perspective, the variables are described using average value or percentages, according to the type of variable. Subsequently, correlations are established and a multiple linear regression is performed. A binary dummy variable is created for inclusion in the study, from a qualitative variable such as the type of entity: Urban, Intermediate and Rural. Decomposing the Rural entity as value “0” in the two dummy variables (URB1 = 0, URB2 = 0), the Intermediate entity has the values (URB1 = 0, URB2 = 1) and the Urban entity has the values (URB1 = 1, URB2 = 0).

## Results

During 2010, 62,804 people experienced an active process of depression and visited their family physician. This supposes a prevalence of 5.67%, with an average age of 59.50 years and with a greater proportion in women (3 women for each man). The female subgroup revealed a progressive and rapid increase in the prevalence curve of depression up to 55 years, then stabilizing and reaching a plateau up to 65 years of age, with abrupt and decrease occurring between 65 and 74 and then having another equally sharp rise until 80. In the male subgroup, there was a slow progressive increase in the number of prevalent cases of depression until the age of 50; there was a tendency to stabilize until the age of 64, only to descend slowly with a small increase until 77–80 years.

Analyzing by BHAs, the depression prevalence obtained is 4.55% (CI 4.20–4.89), with an interquartile range (Q3–Q1) of 2.97 and a range of 8.36. This gives us an idea of the differences found in the prevalence rates throughout the analyzed territory. A maximum value of 8.79% appears in a BHA in the main nucleus of Aragon (Zaragoza), with a lower prevalence of depression outside the main population centers of Aragon.

The correlations between the adjusted prevalence rates of depression by BHA and the analyzed variables are shown in Table 2. The Pearson P statistic was used for the variables over-aging, widowed/separated/divorced, foreigners and middle-school; whereas in the analysis of the rest of the variables, Spearman’s rho statistic has been used since they are ordinal variables or do not follow a normal distribution. It may be observed that the moderate and positive correlations appear with higher rates of depression in areas having a higher femininity index, higher population density, areas with a higher unemployment rate and higher average salary. And a positive weak correlation is seen in relation to the percentage of singles.

**Table 2:** Correlations between the prevalence of depression and the variables studied.

	Correlation rate
*Population distribution*	
Density of population	0.685**
*Typology of entity*	
Rural/intermediate/urban	-0.601**
*Population structure by gender and age*	
Femininity index	0.563**
Dependence index	-0.564**
Over-aging index	
+75	-0.518**
+85	-0.307**
*Population structure by nationality*	
Rate of foreigners	0.085
*Population structure by education*	
Average education degree	0.429**
*Population structure by work*	
Unemployment rate	0.523**
*Population structure by salary*	
Average salary	0.488**
*Population structure by marital status*	
Single	0.318**
Separated, divorced or widowed	-0.036
*Household structure by number of members*	
Unipersonal	-0.203*
5 or more members	-0.366**
*Stage of the buildings*	
Poor or worse state	-0.302**
Bad or worse state	-0.313**

*^∗^*P*-value < 0.05; ^∗∗^*P*-value < 0.001*.

Weak and negative correlations are found in relation to the state of buildings and the household structure according to the number of people. In relation to the category of BHA, depending on whether they are located in an urban, intermediate or rural environment; a moderate negative correlation has been found. This indicates that there are lower rates of depression in rural areas. Negative and moderate correlations have also been found in relation to the dependency rate and the aging index.

In order to predict the depression prevalence rates of a BHA, an explanatory model was developed based on the following variables: urban/intermediate/rural decomposed into Dummy variables; over-aging-75 and percentage of the state of the building in poor or worse condition. Obtaining a square *R* = 0.422, which would mean that the model would explain 42.2% of the variability of depression prevalence rates and with a significant linear relationship in ANOVA with a significance coefficient < 0.001.

**FIGURE 2:**
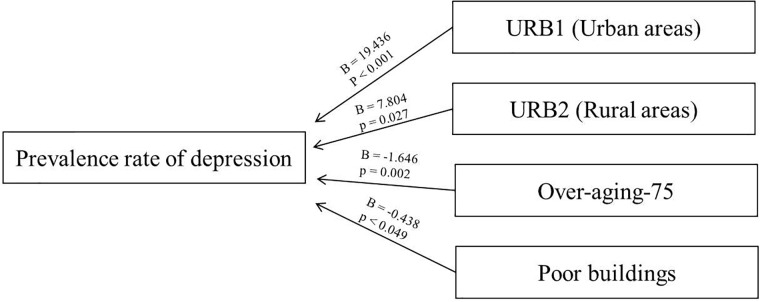
Coefficients related to the multiple linear regression.

The coefficients that would make up the equation: over-aging-75 with a coefficient B = -1.646, meaning that each increase by one unit in over-aging corresponds to a decrease of 1.646 in the prevalence rate of depression. A coefficient of URB1 B = 19.436 and URB2 B = 7.804 for the Urban/intermediate/rural entity of the BHA, which implies greater weight, increasing the pressure prevalence rate if the entity is urban and B = -0.438 for buildings in poor or worse condition. That is, aging +75 and rural entities would act as protective factors for depression, while urban areas and deficient buildings act as risk factors. These relationships are shown in Figure 2.

The equation of the linear regression is as follows: Prevalence rate = 106.96 + 19.436^∗^URB1-1646^∗^Over-aging-75 + 7.804^∗^URB2-0.438^∗^Poor buildings.

## Discussion

This ecological methodology may be a useful tool which, together with the statistical epidemiological analysis, can help in the political decision making process, and in this case, with the specific objective of health and, especially, mental health and depression. Relating diseases with the environment where they are generated may help to improve the health attention in the BHA, since it includes the community care in the day-to-day work. Thus, through a study of correlations, we can observe the association of depression illness with social or demographic determinants. And with a regression model, we can perceive which of the study areas are more likely to have a population that may suffer from depression. Thus, in the predictive regression model of depression, 42.2% of the variability of depression prevalence rates would be explained, whereas the other 57.8% of the variation would be attributable to other causes of a genetic, psychological or family nature. It must be considered that the model shows a key factor such as the type of rural/intermediate/urban environment being an unchangeable factor, so it is up to the public administrations to facilitate dynamics that modify habits, relationships and the day to day of people in the urban environment (Dufouil et al., 1995; Weich et al., 2006). With respect to the variables obtained in the model of over-aging, they should be analyzed with caution in regards to a potential under-diagnosis, and an influence of the rural environment variable, since in the analyzed territory the rural world is very old. The state of the buildings variable could in turn be correlated with over-aging (older people tend to live in old buildings).

If we analyze the obtained results, with respect to the depression rate, we have obtained a total prevalence of depression of 5.67%, which corresponds to a lower prevalence than that offered by the large European studies having a level of 10.55% (ESEMeD in Spain) or the PREDICT study in Spain with a 12.2% depression from primary care consultations (Haro et al., 2006; King et al., 2008). Given that these studies used population samples in which they have passed diagnostic tests and since in this study, the diagnoses were made by the family physician in a real context, the existence of under-diagnosis in Primary Care consultations could be considered in Aragon. This would be in agreement with other studies that affirm that the number of patients with depression diagnosed by the family doctor is between 42 and 72% of the total of depressed patients. Subjects that are not recognized by the family doctor are the least symptomatic (Simon and Von Korff, 1995; Vazquez-Barquero et al., 1997; Simon et al., 1999). The detection is related to the educational level of the patients, the severity of the symptoms, the level of disability and the explicit complaint of psychological depressive symptoms (Aragones et al., 2004).

The infradiagnosis may be influenced by the age of the patients, since there are significant differences when comparing Aragon rates and European rates for adults under and over 65 years; in people older than 65 years, an average that is 4.72 points less than that of European prevalence has been found whereas, on the other hand, it is 2.84 points less for adults under the age of 65. The possible considerable under-diagnosis in the older population is thus reflected in several studies (VanItallie, 2005; Urbina Torija et al., 2007; Kessler et al., 2010). This may be explained by the fact that older people express symptoms of depression as physical ailments or also because they may be confused with symptoms of dementia or long chronic diseases (Gum et al., 2009). But it is also important to analyze the rate of depression prevalence in individuals over 65 while taking account the demographic context, not only adjusting for sex and age, but also for aspects such as rurality or not of populations, because this aspect influences social networks support (Probst et al., 2006).

Regarding the variability obtained in the depression rates in the different BHA in relation to the other environmental factors analyzed, in the correlation analysis obtained, many correlations are supported by the bibliography, but other correlations have also appeared that should be studied in greater depth.

In the anticipated results based on the consulted bibliography, the traditional protective role of the rural environment in mental illnesses has been shown in this study (Vink et al., 2008; McKenzie et al., 2013). Expected results related to higher rates of depression are unemployment (Whooley et al., 2003); the higher population density, which is related to the variable rural/urban environment; and higher rates of femininity, which also correlates with the prevalence rates of depression according to gender (Aragonès Benaiges et al., 2003; Barry et al., 2008); and a lower dependency rate, supported by studies of depression according to the life cycle (Gum et al., 2009; Kessler et al., 2010).

However, as an unexpected result, a higher average salary correlates with an increase in the rate of depression. There are many studies that relate depression to low socioeconomic levels, in fact subjects with a low socioeconomic status have a 2.5 risk of developing depression (Araya et al., 1998; Ostler et al., 2001; Aragones et al., 2004), but the interaction between depression, social classes, and low economic level is complex (Lewis et al., 1998). Some studies reveal that if the degree of social isolation is controlled, the effects of poverty are reduced (Bruce et al., 1991; Bruce and Hoff, 1994) and other studies affirm that the main effect of poverty does not increase the prevalence of mental disorders, but prolongs the duration of the episodes (Weich and Lewis, 1998; Lorant et al., 2007). In this study, we consider that the reason of this unexpected result is the concentration of depression in urban areas, where the inequality is more evident (Fernández Morales, 2003). Anyway following studies should focus in this topic.

Regarding the average education, which it may be considered a protective factor (Koster et al., 2006), as an unexpected result of this study, higher depression rates has been obtained when higher levels of education are achieved. However, when analyzing within the urban environment, it appears that a higher degree of education in a large city exerts a certain protective factor against depression whereas in the intermediate and rural environment, those with higher educational levels encounter worse state of mental health. This may be due to the fact that the work to be done in the intermediate-rural environment does not meet the expectations of the degree that was earned or because it is more difficult to advance in the academic or professional field (Stansfeld et al., 2003; Arslan and Acar, 2013).

There is no significant relationship between depression and the rate of foreigners in this study, as is shown in previous studies that find only a significant relationship in recently immigrated and low-income women (Plant and Sachs-Ericsson, 2004; Smith et al., 2007). There is no significant relationship between depression and the percentage of widowers, separated-divorced, perhaps partly due to the non-disaggregation of data in adequate subgroups. More exhaustive studies with disaggregated data show that the couple’s relationship influences depression rates (Bruce and Kim, 1992; Lorant et al., 2007).

This study has certain limitations that are mainly focused on the data source of the depression rate, since they have been obtained from electronic medical records, and this may be a cause of possible under-diagnosis, especially in those over 65 years. However, it is the data from these electronic medical records that are used in statistical epidemiological analyses and that are also used in decision making on a political scope. Therefore, it is necessary to establish educational and organizational measures in order to correctly diagnose the depression episode, especially in the population group over the age of 65.

## Conclusion

When studying depression in its environment, we obtain an ecological analysis for the rates of depression. The variable found to have the greatest weight in the model obtained from the multiple linear regression is the rural/urban environment. Although this factor is unchangeable on its own, it could be compensated with the establishment of dynamics that modify habits, relationships and the daily life of individuals living in urban environments.

## Ethics Statement

The authors assert that all procedures contributing to this work comply with the ethical standards of the Clinical Research Ethics Committee of Aragón (belonging to the Department of Health of the Government of Aragon, Spain) and with the Helsinki Declaration of 1975, as revised in 2008. The Study Protocol was approved by the Clinical Research Ethics Committee of Aragón (Spain) (10/2008) and by the Aragones Health Service (2009). Data of prevalence of depression were obtained from clinical records provided in a non-identifiable format by Aragones Health Service.

## Author Contributions

JL, MZ-A, and RM-B were responsible for the conception, data collection, and design of the study. JL and EA were responsible for data analysis. JL, BO-B, BM, RM-B, and JC contributed to the interpretation of data. JL, MZ-A and BO-B wrote the article, which was critically revised by all the other authors. All authors have approved the final version of the manuscript.

## Conflict of Interest Statement

The authors declare that the research was conducted in the absence of any commercial or financial relationships that could be construed as a potential conflict of interest.
